# Advancing microbial engineering through synthetic biology

**DOI:** 10.71150/jm.2503100

**Published:** 2025-03-28

**Authors:** Ki Jun Jeong

**Affiliations:** 1 Department of Chemical and Biomolecular Engineering, KAIST, Daejeon 34141, Republic of Korea; 2 Graduate School of Engineering Biology, KAIST, Daejeon 34141, Republic of Korea

Synthetic biology has transformed microbial engineering, enabling precise genetic modifications for diverse applications, from biopharmaceutical production to environmental sustainability. Unlike traditional methods, which relied on labor-intensive and unpredictable optimization, synthetic biology offers scalable and modular design principles, enabling the rational engineering of microbes. Advances in genome editing, metabolic pathway design, and synthetic regulatory networks have improved strain development, significantly enhancing microbial productivity and robustness. CRISPR-based genome engineering, for instance, allows precise modifications to specific genes, fine-tuning microbial performance. Coupled with machine learning and high-throughput screening, these tools accelerate microbial optimization, increasing yields in biofuel, pharmaceutical, and chemical production. Engineered microorganisms, such as *Escherichia coli* and *Saccharomyces cerevisiae*, as well as non-model organisms, are being developed with modular genetic components, facilitating customized strain design for various industrial needs.

Now, synthetic biology is making significant strides across multiple sectors. In healthcare, engineered microbes produce therapeutic proteins and vaccines efficiently and cost-effectively. Microbiome engineering and synthetic microbial consortia are also driving innovations in personalized medicine. In manufacturing, synthetic microbes are replacing petrochemical-based production with bio-based alternatives, generating bioplastics, biofuels, and biodegradable materials, thus reducing environmental impact. Furthermore, these microbes are being utilized for bioremediation, helping to mitigate climate change effects. In agriculture, synthetic biology enhances crop resilience and soil health, contributing to sustainable farming practices. It also plays a role in food production, developing alternative proteins and fermentation-based substitutes, promoting more sustainable and ethical food systems.

This special issue aims to showcase the latest advancements in the field, providing insights into the transformative potential of synthetic biology. It includes eight reviews on the recent development of synthetic biology tools for genome editing, fine tuning of gene expression, and genetic code expansion and also reviews on recent application to microbial cell factories including *Corynebacterium glutamicum*, methanotrophs, yeast and microalgae.

Kim et al. (2025) discussed recent advancements in targeted mutagenesis, focusing on three key approaches: orthogonal error-prone DNA polymerases, site-specific base editors, and homologous recombination with mutagenic DNA fragments. These technologies enable efficient, continuous mutagenesis with high-throughput screening, reducing labor and time. Despite advancements, challenges remain in enhancing mutation rates, reducing off-target effects, and expanding mutation windows. The review emphasizes the need for tailored strategies depending on specific applications, such as enzyme improvement, metabolic engineering, and drug resistance. The integration of these tools with synthetic biology promises breakthroughs in protein engineering and industrial biotechnology.

Ren et al. (2025) discussed the importance of untranslated regions (UTRs) in optimizing bacterial gene expression for various biotechnological applications. It highlights strategies such as AU-rich elements, G-quadruplex structures, synthetic dual UTRs, ProQC systems, and riboswitches for fine-tuning gene expression, improving translation fidelity, and enabling dynamic regulation. The integration of UTR-based tools offers a versatile approach to enhance metabolic engineering and synthetic biology. Additionally, the use of predictive computational tools can accelerate the rational design of UTR elements, expanding their potential in biotechnology, industrial bioproduction, and therapeutic applications.

Lee et al. (2025a) discussed advancements in genetic code expansion, specifically the incorporation of non-proteinogenic monomers (npMs) into proteins in vivo. It highlighted the use of engineered translation systems, such as aaRS/tRNA pairs, to incorporate diverse npMs. The review also emphasized translation-independent methods like tREX, bio-mREX, and START, and the potential of computational approaches for refining genetic components. Future directions include optimizing microbial platforms for non-natural polymer production and developing novel enzymes for innovative catalytic reactions.

Jeon et al. (2025) explored the transition from traditional metabolic engineering to systems metabolic engineering, with a focus on the Design-Build-Test-Learn (DBTL) cycle for developing optimized microbial strains. It highlighted the case of *Corynebacterium glutamicum*, a well-established amino acid producer that has been engineered to produce high-value C5 chemicals, such as 5-AVA, GTA, and 1,5-PDO, by leveraging systems metabolic engineering approaches. The review emphasized the potential of systems metabolic engineering as a more eco-friendly and sustainable alternative to traditional petrochemical processes. It also discussed the importance of bridging systems biology insights with experimental findings and refining the DBTL cycle to address uncertainties and improve strain development. Ultimately, systems metabolic engineering is poised to drive innovation and contribute to the sustainable production of diverse biochemicals in industrial biotechnology.

Lee et al. (2025b) highlighted the potential of methanotrophs as efficient biocatalysts for methane bioconversion, offering advantages over energy-intensive chemical processes. Methanotrophs utilize methane as their sole carbon and energy source, enabling the production of value-added compounds such as methanol, organic acids, ectoine, and polyhydroxyalkanoates. Over the past decade, advances in genome sequencing, transcriptomics, and genetic engineering have deepened the understanding of methanotroph metabolism and enabled the development of engineered strains for enhanced bioconversion. This review provided an overview of methanotroph metabolic pathways and recent progress in metabolic engineering, emphasizing breakthroughs in strain development. By summarizing key developments, the review identified both opportunities and challenges in scaling up methane bioconversion technologies for industrial applications, offering insights into how future research can address existing limitations.

Tran and Lee (2025) explored organelle engineering as a strategy to enhance microbial biosynthesis in yeast. Conventional metabolic engineering in the cytoplasm faces challenges such as metabolic crosstalk, competing pathways, and precursor limitations. To overcome these issues, recent studies have repurposed organelles like mitochondria, peroxisomes, and the endoplasmic reticulum as specialized microbial factories. Key strategies include engineering signal peptides, optimizing cofactors and energy supply, enhancing organelle biogenesis, and dual organelle engineering. Finally, the review discussed the potential and challenges of organelle engineering and proposes an automated pipeline to maximize its capabilities for biofuel and biochemical production in yeast.

Le et al. (2025) discussed the potential of yeast cell factories, particularly *Saccharomyces cerevisiae* and *Yarrowia lipolytica*, for producing biodegradable plastics like PLA, PHAs, and PBAT. It highlighted advancements in synthetic biology, including metabolic engineering, protein engineering, and adaptive evolution, to enhance strain efficiency and product yields. The review also emphasized integrating computational tools and machine learning into the Design-Build-Test-Learn cycle to improve strain development, minimize effort, and overcome challenges in strain robustness and scaling. By combining synthetic biology with computational approaches, yeast-based systems offer significant potential for sustainable, scalable bioplastic production.

Tran et al. (2025) focused on the use of CRISPR/Cas systems for precise genome editing in microalgae, highlighting advancements in improving transformation efficiency, gene editing precision, and overcoming species-specific challenges. It discussed the use of Cas9, Cas12a, and other CRISPR-based approaches to enhance microalgal biotechnology, particularly in biofuel and biomolecule production. Despite challenges such as interspecies genetic variation and off-target effects, efforts to optimize CRISPR components are unlocking new possibilities for metabolic engineering. The review emphasized the need for further research to improve understanding of genetic mechanisms and enable large-scale applications in microalgal synthetic biology.

Despite recent advances in synthetic biology field, challenges remain, including the complexity of biological systems and ensuring the stability of engineered microbes in industrial settings. Ethical and regulatory concerns also require careful consideration to ensure responsible deployment. Future innovations will focus on integrating artificial intelligence, automation, and synthetic biology, enabling the creation of self-regulating microbial systems for more efficient and sustainable bioproduction. The convergence of synthetic biology and microbial engineering is unlocking new possibilities for biotechnology and industry. By overcoming existing challenges and leveraging interdisciplinary approaches, synthetic biology will continue to revolutionize microbial engineering, driving innovations that benefit society and the environment. As we enter a new era of bio-based solutions, the collaborative efforts of researchers, industry leaders, and policymakers will be key to ensuring the responsible and impactful development of synthetic biology-driven microbial technologies.
